# Current Status and Evolution of Preclinical Drug Development Models of Epithelial Ovarian Cancer

**DOI:** 10.3389/fonc.2013.00296

**Published:** 2013-12-11

**Authors:** Panagiotis A. Konstantinopoulos, Ursula A. Matulonis

**Affiliations:** ^1^Medical Gynecologic Oncology Program, Department of Medical Oncology, Dana Farber Cancer Institute, Harvard Medical School, Boston, MA, USA

**Keywords:** epithelial ovarian cancer, high-grade serous, preclinical models, personalized therapy, cell lines, xenografts, mouse models, patient-derived xenografts

## Abstract

Epithelial ovarian cancer (EOC) is the most lethal gynecologic malignancy and the fifth most common cause of female cancer death in the United States. Although important advances in surgical and chemotherapeutic strategies over the last three decades have significantly improved the median survival of EOC patients, the plateau of the survival curve has not changed appreciably. Given that EOC is a genetically and biologically heterogeneous disease, identification of specific molecular abnormalities that can be targeted in each individual ovarian cancer on the basis of predictive biomarkers promises to be an effective strategy to improve outcome in this disease. However, for this promise to materialize, appropriate preclinical experimental platforms that recapitulate the complexity of these neoplasms and reliably predict antitumor activity in the clinic are critically important. In this review, we will present the current status and evolution of preclinical models of EOC, including cell lines, immortalized normal cells, xenograft models, patient-derived xenografts, and animal models, and will discuss their potential for oncology drug development.

## Introduction

Epithelial ovarian cancer (EOC) is the most lethal gynecologic malignancy and the fifth most common cause of female cancer death in the United States (1). Advanced stage at diagnosis for most women with this cancer and emergence of resistance to conventional chemotherapy are primarily responsible for this dire outcome. Although important advances in surgical and chemotherapeutic strategies over the last three decades have significantly improved the quality of life and median survival of EOC patients, the overall cure rate has not improved appreciably (2–5). EOC is a genetically and biologically heterogeneous disease and is traditionally divided into two types (types I and II) with distinct genotypic and phenotypic characteristics which are summarized in Table 1 (6–8). Type I tumors frequently harbor somatic mutations in KRAS, BRAF, PIK3CA, PTEN, CTNNB1, and ARID1A genes, and exhibit low genomic instability without genome-wide copy number changes (9) while type II tumors are characterized by high degree of genomic instability with high frequency of DNA copy number changes and p53 mutations (6, 7, 10).

**Table 1:** **Molecular and clinical characteristics of EOC subtypes**.

Histology	Type	Molecular characteristics	Clinical characteristics
Low grade serous carcinoma	I	KRAS, BRAF mutations	Frequently arise from serous cystadenoma-borderline sequence
			Relatively indolent growth
			Poor response to platinum based chemotherapy
Low grade endometrioid carcinoma	I	CTNNB1, PTEN, PIK3CA, and KRAS mutations	Frequently arise from endometriosis
		Microsatellite instability	Relatively indolent growth
			Association with HNPCC^a^
			Poor response to platinum based chemotherapy
Clear cell carcinoma	I	PIK3CA, ARID1A mutations	May arise from endometriosis
		MET amplification	Association with HNPCC^a^
			Worse prognosis and response to platinum based chemotherapy
Mucinous carcinoma	I	KRAS mutations	May arise from cystadenoma-borderline sequence
		HER2 amplification
High-grade serous and high-grade endometrioid carcinoma	II	P53 mutations (almost universal), BRCA1, BRCA2 mutations	May arise from fallopian tube intraepithelial carcinoma (TIC)Association with HBOC^b^
		Genomic instability and very high degree of somatic copy number alterations	
			Rapid growth
			Very good response to platinum based chemotherapy

*^a^ HNPCC, hereditary non-polyposis colorectal cancer syndrome due to germline mutations in mismatch repair genes*.

*^b^ HBOC, hereditary breast ovarian cancer syndrome due to germline BRCA1 or BRCA2 mutations*.

High-grade serous carcinomas (HGSCs) represent the most common type II histologic subtype and account for approximately 70% of all EOCs. These tumors exhibit histological features that are identical to those of primary peritoneal and fallopian tube serous cancers and are treated similarly to these neoplasms. A number of molecular studies and most recently The Cancer Genome Atlas (TCGA) project have shown that HGSCs are characterized by frequent genetic and epigenetic alterations in gene members of the homologous recombination (HR) DNA repair pathway, including the BRCA1 and BRCA2 genes (10). Furthermore, the NOTCH, FOXM1, RB, and PI3K/RAS signaling pathways have also been implicated in the pathogenesis of HGSCs (10). These important advances in our understanding of the molecular pathogenesis and heterogeneity of EOC hold promise for the development of novel therapies against these tumors. However, for this promise to materialize, appropriate preclinical experimental platforms that recapitulate the complexity of these neoplasms and reliably predict antitumor activity in the clinic are critically important. In this review, we will discuss the current status and evolution of preclinical models of EOC focusing on their potential for oncology drug development.

## Cell Lines

Historically, ovarian cancer cell lines have been the most frequently used tumor models to prescreen experimental anticancer agents *in vitro* and to select specific histologic subtypes of EOC for further exploration of these agents. These cell lines have undergone a high degree of evolutionary selection pressure *in vitro* as they have been in passage for several years (or even decades in some cases). As a result, their genomic profiles have been irreversibly altered and rarely recapitulate the genetic and pathologic characteristics of the parental cells (11–13). Furthermore, cancer cell lines lack the molecular heterogeneity of the parental tumor and are molecularly skewed toward affinity to grow in monolayers.

In a recently published study, Domcke and colleagues used available molecular profiles (copy number changes, mutations, and mRNA expression profiles) of cell lines from the Cancer Cell Line Encyclopedia (CCLE) and of tumor samples from the TCGA to evaluate the suitability of 47 EOC cell lines as *in vitro* models of HGSCs (14). The investigators showed significant differences in the molecular profiles between commonly used EOC cell lines and HGSC samples and reported that the presumed histologic subtype for several of these cell lines did not correspond to their molecular profiles. Of note, the two most frequently used cell lines, SKOV3, and A2780 were deemed unsuitable as HGSC models, while other rarely used cell lines such as KURAMOCHI, OVSAHO, and SNU119 closely resembled the molecular profiles of HGSC samples. Interestingly, the suitability of these cell lines as HGSC models did not correlate with time of their derivation suggesting that number of passages may not correlate with model suitability. Among the cell lines deemed most suitable to use as HGSC models, three cell lines harbored BRCA mutations i.e., KURAMACHI (BRCA2), COV362 (BRCA1), and JHOS2 (BRCA1) and therefore may be useful as *in vitro* models for BRCA-associated EOC.

This study may provide molecular explanation for the challenges of translating preclinical observations from ovarian cancer cell lines into the clinic, a problem that is not unique to ovarian cancer but transcends multiple tumor types (14, 15). However, this study also highlights that certain EOC lines may still hold value as HGSCs models and underscores the importance of evaluating and screening them to confirm their origin and molecular resemblance with HGSC. This is now feasible given the increasing availability of large scale genomic data from studies such as the TCGA, the CCLE, and the Sanger Cancer Cell Line project (10, 16). Cell line models whose molecular identity has been confirmed using targeted sequencing and copy number profiling may be extremely valuable as preclinical models, particularly those with well defined molecular alterations such as BRCA1/2 or PI3K mutations in order to assess the potential of experimental drugs in patient populations with specific molecular alterations. In this regard, the promise of PARP inhibitors in the management of BRCA-deficient EOC was first realized in BRCA1/2 deficient cell lines (17, 18). In the era of advanced molecular profiling, using cell lines with molecular similarities with patient samples may increase the possibility that *in vitro* observations will be eventually translatable to the clinic.

## Immortalized Normal Cells and Stem Cells

Several investigators have reported isolation, *in vitro* propagation and immortalization of human ovarian surface epithelial (OSE) and fallopian tube epithelial (FTE) cells which are considered the cells of origin of ovarian carcinomas. Retroviral transduction of either the human papilloma virus E6/E7 oncogenes or the simian virus 40 T-Antigen (SV40-TAg) in human OSE cells leads to increased and sustained proliferation even after multiple passages but does not induce transformation (19, 20). For immortalization to occur, additional retroviral constructs targeting TP53, hTERT, or RB are required (21, 22). Besides retroviral transduction, RNA interference technology has been successful in immortalizing human OSE cells as exemplified by the work of Yang and colleagues who successfully immortalized OSE cells via siRNA knockdown of p53 and Rb (23, 24). As with human OSE cells, Karst and colleagues immortalized normal human FTE cells via retroviral transduction of hTERT and either of SV40-TAg or an shRNA targeting p53 and mutant CDK4^R24C^, while transformation occurred via further ectopic expression of either MYC or HRAS oncogenes (25). When injected in immunocompromised mice, these cells developed tumors resembling HGSCs both histologically and clinically. Shan and colleagues used a similar approach of hTERT and SV40-TAg overexpression for immortalization and of additional ectopic HRAS expression for transformation of human FTE cells while similar results have been reported by Jazaeri and colleagues (26, 27).

Although presence of ovarian cancer stem cells has been reported, definite characterization of these cells is still lacking (28). Furthermore, the stem cell niche of the OSE which regenerates after each ovulation has not been determined. There have been several reports of ovarian cancer stem cells isolation which have been based on markers and protocols used to define stem cells in other tumors including leukemia, colon, and breast cancers (29–31). In a seminal study, Flesken-Nikitin and colleagues proposed that the hilum region of the mouse ovary is a stem cell niche of the OSE (32). Specifically, the investigators showed that hilum cells express stem cell markers ALDH1, LGR5, LEF1, CD133, and CK6B, display long-term stem cell properties *ex vivo* and *in vivo* and exhibit increased transformation potential after inactivation of TP53 and RB1.

## Xenografts

Xenograft models have been extensively used in ovarian cancer research and are still very important experimental platforms for preclinical drug development (33–36). These models require use of immunodeficient mice strains, i.e., athymic nude mice lacking T lymphocytes, severe combined immunodeficient (SCID) mice which lack functional B and T lymphocytes, or the NOD/SCID/IL2Rγ^null^ mice which also exhibit inactive innate immunity due to abrogation of maturation of natural killer (NK) T cells (37). The requirement of immunodeficiency has often been cited as one of the main reasons why xenografts have shown limited predictive value in the clinic (38, 39). Specifically, tumor xenografts in immunocompromised mice cannot recapitulate either the contributions of immune factors on tumor development and progression or the extensive interactions of the human host tumor microenvironment (stroma, extracellular matrix, and vasculature) with the tumor cells.

Traditionally, xenograft models rely on implantation of established EOC cell lines subcutaneously, intraperitoneally, or orthotopically. Subcutaneous implantation offers the advantage of easy quantification of tumor volume which is ideal for assessing antitumor efficacy of experimental agents, but rarely results in ascites formation or intraperitoneal (IP) seeding of the tumor, and thereby fails to reflect the clinical course of human EOC. Conversely, IP and orthotopic implantation (OI) frequently result in peritoneal carcinomatosis and development of malignant ascites. The most commonly used xenograft model in ovarian cancer was developed by IP injection of a subpopulation of the drug resistant cell line NIH:OVCAR-3 (40) (isolated by serial *in vitro* and *in vivo* selection of cells) into athymic mice which resulted in development of ascites and peritoneal carcinomatosis (33). The NIH:OVCAR-3 cell line has been molecularly ranked as possibly of HGSC origin on a rank of likely, possibly and unlikely, and this xenograft model is still widely used today (14). The OVCAR-3 and other xenograft models have been used in the preclinical evaluation of antiangiogenic agents (41, 42). Specifically, these models demonstrated the ability of a monoclonal antibody (mAb) to human vascular endothelial growth factor (VEGF) to prevent ascites formation and that combination therapy with inhibitors of VEGF plus paclitaxel exhibits synergistic reduction of tumor growth and ascites in ovarian cancer. These observations were subsequently confirmed in clinical trials of bevacizumab as single agent and in combination with paclitaxel in EOC (43–45).

Orthotopic implantation involves injecting EOC cells into their natural position adjacent to the ovaries which in mice corresponds to the ovarian bursa, a thin membrane that encapsulates the ovaries (46). OI is usually accomplished by direct injection within the ovarian bursa via the infundibulum (47, 48). OI recapitulates initiation of EOC growth in the ovaries, does not require selection of EOC cell lines, and preserves tumor histology and the potential for peritoneal dissemination and ascites formation. Furthermore, several studies have indicated increased tumor take rates with OI thereby reflecting a more favorable microenvironment for tumor growth and metastatic dissemination (48, 49). Unlike subcutaneous xenografts, orthotopic and IP xenografts pose a challenge for accurately quantifying tumor volume and monitoring disease progression thus making them less appealing as models for preclinical drug development. However, this challenge may be overcome by advances in non-invasive imaging of tumors in mice [magnetic resonance imaging (MRI), ultrasound (US), positron emission tomography (PET), computed tomography (CT), and single photon emission computed tomography (SPECT)] and/or use of fluorescent or bioluminescent reporters with optical imaging [fluorescent imaging (FLI) or bioluminescent imaging (BLI)] and/or use of serum tumor biomarkers such as CA125 (50).

## Patient-Derived Xenografts

Patient-derived xenografts (PDXs) represent an evolution of the cell line xenograft model whereby fresh tumor tissue, obtained directly from patients, is implanted subcutaneously or orthotopically into immunodeficient mice (51, 52). After a variable period of time, PDXs enter a logarithmic growth phase which allows for harvesting and reimplantation in successive mice generations with reported tumor engraftment rates higher than 75% (53–55). The time to engraftment depends on the individual tumor, the site of implantation and the type of immunodeficient mice used (NOD/SCID/IL2Rγ^null^ mice are associated with superior engraftment efficiency) and is generally between 2 and 4 months. PDXs have been successfully established from primary or metastatic tumors (56, 57), from untreated or heavily pretreated tumors (58, 59) thereby potentially capturing chemotherapy-refractory tumor populations and permitting the study of molecular changes that occur at the time of development of resistance.

A growing body of literature suggests that PDXs hold significant promise as models for preclinical drug development because they closely resemble and recapitulate tumor growth in humans (Table 2). In a seminal study by Hidalgo and colleagues, the investigators treated PDXs from 14 patients with various advanced solid tumors with 63 drugs in 232 treatment regimens, and showed that there was an excellent correlation between response in the PDX models and patient response to these regimens (60). Of note, in some cases, the treatment administered to patients based on the PDX response was not the first choice of the oncologist treating these patients. This study highlights the potential of PDXs as experimental platforms for preclinical drug development. PDXs represent significant improvement over the standard cell line xenografts because they maintain the principal characteristics of the original patients’ tumors including histology, mutational status, DNA copy number changes, gene-expression patterns and clinical behavior while they remain biologically stable when passaged in mice. Specifically, genome-wide expression analysis in non-small cell lung cancer has demonstrated that PDXs exhibit similar gene-expression profiles and maintain the key gene and pathway activity of the primary tumors (61). Furthermore, mutational and expression analysis in pancreatic PDXs has shown that there is excellent concordance between primary tumors and PDX models (62). Several studies have also shown that PDXs maintain their molecular similarity (histology, protein expression, tumor biomarkers, genomic, and genetic status) with the primary tumors during sequential passage (63–65). This molecular similarity is even higher when PDX models are generated using patient tumors that are immediately implanted into immunocompromised mice without an intermediate *in vitro* culture step (66, 67). Another key feature of PDXs is the maintenance of the original tumor architecture and histopathological characteristics, including a component of human stroma as well as tumor microvasculature although there is a controversy over how long this is maintained. Specifically, in one study of pancreatic PDXs, vessels with human endothelial cells were maintained or even increased over time while in a similar study with renal cell cancer PDXs, a decrease in human-derived tumor microvasculature was observed (68, 69). Of note, maintenance of human tumor-associated leukocytes including memory T cells for up to 9 weeks after implantation has been reported in lung cancer PDXs implanted into NOD/SCID/IL2Rγ^null^ mice. Furthermore, preservation of pluripotent CD133+ stem cells in PDXs following repeated orthotopic subtransplantations has been reported and in these studies the CD133+ cells continued to exhibit multi-lineage differentiation capacity *in vitro* (70–73). PDXs (particularly early passage PDXs) may therefore be excellent preclinical platforms to study stromal-tumor interactions and cancer stem cell biology as well as to assess novel anticancer agents or drug combinations.

**Table 2:** **Advantages and disadvantages of PDX models**.

Advantages	Disadvantages
Unlike cell lines, PDXs do not undergo evolutionary selection pressure from *in vitro* culture	Immunocompromised mice cannot adequately capture the intact human immune component of primary tumors and thus may not recapitulate the complex cross talk between tumor cells and the human immune system
PDXs maintain the characteristics and heterogeneity of the original tumor i.e., histology, mutational status, DNA copy number changes and gene expression	Human stroma is eventually replaced by murine stroma thereby limiting the ability to recapitulate tumor-stroma interactions in late passages PDXs
PDXs maintain their molecular similarity with the primary tumors during sequential passage	Orthotopic implantation is technically challenging
PDXs include a component of the primary tumor’s stroma including microvasculature, stem cells, and memory T cells, although it is unclear for how long this is maintained	Expensive to establish and maintain PDX banks thus requiring significant funding resources or institutional support
PDXs offer the opportunity to evaluate tumors from metastatic sites or tumors that have developed resistance to multiple treatments	Establishment of PDX banks requires prompt processing of primary tumor and significant coordination between departments
Studies have shown very good correlation between response in PDX models and clinical response in patients	Possible regulatory challenges i.e., IRB approval and HIPPA and intellectual property issues

Several limitations of PDXs exist (Table 2). A major limitation of PDXs is the requirement to use immunodeficient mice which limits the number of drugs that can be evaluated (i.e., alternative models are necessary for immune-modulating agents) (74, 75). Furthermore, severely immunocompromised mice cannot adequately capture the intact human immune component of the primary tumors and thus may not recapitulate the complex cross talk between tumor cells, stroma, and the human immune system. One approach to circumvent this problem may be transplantation of human CD34+ cord blood cells enriched for human hematopoietic stem cells that may reconstitute a human innate and adaptive immune system in mice (76). However, development of PDX models in mice with a reconstituted human immune system is technically challenging and would require that the xenografted tumors and the human immune cell component are HLA matched. Furthermore, the eventual replacement of human stroma by murine stroma is an important disadvantage of PDX models given the importance of tumor-stroma interactions in mediating drug response and resistance. Therefore drugs that target the tumor-stroma or microvasculature such as antiangiogenic agents may also require alternative models for evaluation. Murine models are also known to be imperfect models of drug metabolism and distribution in humans. For example, an overestimation of response may occur when drugs are tolerated at higher doses in mice while an underestimation may occur when mice are less tolerant to drugs compared to humans. There also several logistic challenges including financial and personnel resources that are necessary to establish and maintain PDX banks and the ability to freeze and reestablish tumors after months of storage. Compared to the inexpensive cell line experiments, the cost burden of PDX tumor models is substantial and will likely require significant institutional and national funding to support widespread use of PDXs as experimental models.

In EOC, Kolfschoten and colleagues have reported development of a panel of 15 human ovarian cancer xenografts (12 from fresh tumor derived from patients and 3 from EOC cell lines) grown subcutaneously in the flank of athymic nude mice (77). They assessed the sensitivity of these xenografts to six commonly used anticancer agents and showed that their panel reflected the response rates known for similar drugs in ovarian cancer patients. This study, together with several analogous studies in other tumor types, suggests that PDXs may be used for drug screening in EOC. In our institution, in collaboration with the Belfer Institute of Applied Cancer Research we have embarked on building a platform of ovarian cancer PDXs. The goal of this project is to provide a resource for evaluating efficacy of experimental agents and to identify novel predictive and pharmacodynamic biomarkers. Ovarian cancer cells taken from consented patients are implanted intraperitoneally into immunodeficient mice and these tumors grow and disseminate in the peritoneal cavity similar to human EOC (manuscript in progress, personal communication, Joyce Liu). In order to accurately quantify tumor growth and assess response to experimental therapies, ovarian cancer cells derived from the initial passages are tagged with luciferase and reimplanted into mice for non-invasive BLI. In addition, surrogate biomarkers such as CA125 are evaluated in each of the models to monitor response to therapy.

In the era of personalized medicine, patient-centric PDX models for tumor growth and assessment of drug efficacy may be a valuable resource for the preclinical development of experimental anticancer agents. However, as in the case of cell lines, periodic molecular assessment of these models examining the fidelity to the patients’ original tumors in terms of genetics and histology, two factors that are major determinants of their eventual predictive ability.

## Animal Models

Spontaneous EOC models including the aging hen, the cynomolgus macaque, and the rhesus macaque are rarely used in preclinical drug development due to their low incidence rates and long interval until cancer development (78–80). However, because of its anatomic resemblance to humans, the cynomolgus macaque has been occasionally used to evaluate novel agents such as chimeric antibodies or antibody-cytotoxic conjugates (81, 82). Similar to spontaneous EOC models, chemically or hormonally induced models of EOC are rarely used because their histopathological features are not always predictable and their individual molecular alterations are not well defined (83). Conversely, genetically engineered animal models may be promising platforms for preclinical drug development and will be reviewed below (48, 84).

### Virus-mediated gene delivery

The first successful mouse model of EOC using a retroviral gene delivery system was reported in 2002 by Orsulic and colleagues (85) who isolated OSE cells from transgenic mice which carried the avian tumor virus receptor A (TVA) under the transcriptional control of the b-actin or keratin 5. Using this TVA retroviral delivery system, they infected OSE cells from TVA; p53^−/−^ mice with any combination of two or three of the c-MYC, KRAS, and AKT oncogenes, and reimplanted them in the TVA; p53^−/−^ mice resulting in rapid formation of tumors 8 weeks later. The resulting tumors exhibited poorly differentiated histology with areas of papillary structures resembling HGSCs. This model was subsequently used to assess sensitivity to molecular pathway inhibitors; for example tumors with AKT and c-MYC oncogenes or AKT and KRAS were sensitive to mTOR inhibitor rapamycin while tumors with all three oncogenes (KRAS, c-MYC, and AKT) were resistant to rapamycin but sensitive to a combination of mTOR inhibitor and MEK inhibitor (i.e., rapamycin and PD98059). These experiments highlight how such models may be used to test the efficacy of molecular targeted agents in EOC. A similar experimental strategy was also employed for development of a BRCA1-associated EOC model whereby expression of c-MYC resulted in transformation of BRCA1 and p53 deficient murine OSEs (86). When implanted intraperitoneally in mice, these cells developed tumors with several characteristic of BRCA1-associated HGSCs, i.e., papillary architecture, peritoneal carcinomatosis, development of malignant ascites, and enhanced sensitivity to cisplatin.

### Transgenic models

A transgenic EOC model was developed by Connolly and colleagues (87) by expressing the early region of SV40-TAg under the transcriptional control of Mullerian Inhibitory Substance Receptor II (MISRII). Fifty percent of the transgenic founder mice developed very aggressive tumors (poorly differentiated carcinomas with rapid development of peritoneal carcinomatosis and ascites) but none of them were fertile. In a subsequent report (88), the same group reported a stable transgenic line from a male transgenic founder (TgMISRII-Tag-DR26) whereby all female offsprings developed bilateral EOCs resembling HGSCs. This is the first transgenic model of HGSC and it has been used for evaluation of experimental agents in clinical trials (89).

### Conditional models

Genetically engineered mouse models using conditional expression of tumor suppressor genes via Cre-recombinase-mediated excision of LoxP flanked sequences have been reported extensively in ovarian cancer literature. Given that there are currently no transgenic mice that express Cre-recombinase only in ovarian epithelial cells, localized delivery of recombinant adenovirus expressing Cre-recombinase in the ovarian bursa of mice is required to achieve Cre-LoxP-mediated gene inactivation solely in the ovarian epithelium. Flesken-Nikitin and colleagues (90) first reported intrabursal administration of Ad-Cre for conditional inactivation of p53 and Rb in p53^LoxP/LoxP^; Rb^LoxP/LoxP^ mice which resulted in ovarian tumor formation in 97% of them (39% low grade serous, 45% poorly differentiated, and 15% undifferentiated carcinomas). Peritoneal carcinomatosis and ascites were present in 27 and 24% of the cases respectively. Dinulescu and colleagues (91) developed the first model of endometrioid EOC by conditional expression of an activating KRAS mutation and inactivation of PTEN via intrabursal administration of Ad-Cre in LoxP-Stop-LoxP-KRAS^G12D/+^; PTEN^LoxP/LoxP^ mice. Endometrioid EOCs developed in all mice as early as 7 weeks after injection and were associated with ascites, peritoneal carcinomatosis, and lymph node involvement. Endometrioid EOCs also developed in PTEN^LoxP/LoxP^; APC^LoxP/LoxP^ mice after conditional inactivation of PTEN and APC using intrabursal injection with Ad-Cre (92). These tumors had short latency, 100% penetrance and were associated with peritoneal carcinomatosis and ascites in 21 and 76% of the cases. Importantly, the gene-expression profiles of these tumors closely resembled those of human endometrioid EOCs, particularly those with mutations in the Wnt/b-catenin and PI3K/PTEN pathways suggesting that these models may be promising preclinical experimental platforms for evaluation of novel anticancer agents for these tumors. Another conditional model was reported by Kinross and colleagues (93) whereby intrabursal administration of Ad-Cre for conditional activation of the PI3KCA-H1047R mutation and inactivation of PTEN resulted in ovarian serous adenocarcinomas and granulosa cell tumors.

Finally, a HGSC model was reported by Kim and colleagues (94) by conditionally deleting DICER, a key gene for microRNA synthesis, and PTEN using anti-Mullerian hormone receptor type 2-directed Cre (Amhr2-Cre). HGSCs developed from the fallopian tube in DICER^LoxP/LoxP^; PTEN^LoxP/LoxP^; Amhr2^cre/+^ mice and spread to encapsulate the ovaries and then metastasize throughout the abdominal cavity killing all mice by 13 months. These fallopian tube HGSCs exhibited molecular similarity with human high-grade serous ovarian cancers suggesting that they may be used as preclinical models for drug development. Interestingly, removal of fallopian tubes but not of the ovaries prevented cancer formation confirming the fallopian tube origin of these cancers and providing further support to the hypothesis that the fallopian tube is the primary origin of high-grade serous ovarian cancer (95).

### Limitations of animal models for preclinical evaluation of experimental agents

Although certain genetically engineered mouse models of EOC mimic the origin, histopathology, clinical behavior (peritoneal carcinomatosis, ascites formation, lymph node involvement, and sensitivity to platinum), and molecular fingerprints (gene-expression profiling and mutational events) of EOC, there are several limitations of these models particularly relevant to their use for preclinical evaluation of novel anticancer agents (84). The most significant challenge is the species-specific differences between humans and mice. Telomerase is active in most mouse cells (unlike human cells where it is inactive) and therefore mice tumors require fewer genetic alterations for malignant transformation compared to human tumors. Mouse telomerase activity prevents adequate modeling of the genomic instability of human tumors, particularly of HGSCs which are characterized by high degree of genomic instability. Furthermore, fundamental differences in drug metabolism (protein binding, metabolic rate, and pathways of metabolism) between mice and humans represent a major challenge when mouse models are used for preclinical testing.

Another issue is that mouse models rely on specific oncogenes and tumor suppressor genes while ignoring other aspects of tumor development such as the host immune system and the tumor microenvironment. Due to the limited number of genetic alterations that induce the development of mouse tumors, mouse models are relatively homogeneous and thus may not adequately recapitulate the significant molecular heterogeneity of human tumors which is an essential element of a good preclinical model. Finally, logistical issues including cost, technical challenges in generating GEM models especially GEMs with multiple genetic alterations, long interval until development of tumors and variable penetrance are important limitations of GEM models for preclinical evaluation of novel anticancer drugs.

## Conclusion

Despite significant advances in surgical and medical management, EOC remains a highly lethal malignancy for which new therapeutic strategies are urgently needed. Appropriate experimental platforms that recapitulate the complexity of these tumors are critically important for evaluation of novel therapeutics. Table 3 presents the cell/animal models used for preclinical evaluation of selected experimental agents in EOC and shows the outcome of clinical phase II/III evaluation of these agents. In the first two cases (antiangiogenic agents and PARP inhibitors), cell lines and xenograft models successfully predicted the activity of these agents in phase II/III clinical trials, while in the case of anti-CA125 antibodies and anti-HER-2 agents, preclinical evaluation did not correlate with their phase II/III evaluation. These examples highlight the challenges of preclinical evaluation of novel agents in EOC and underscore the need for appropriate preclinical platforms for a wide variety of experimental agents, i.e., immunotherapies, targeted agents, etc.

**Table 3:** **Preclinical evaluation of selected experimental agents used against EOC**.

Agents	Preclinical models	Reference	Comments
Antiangiogenic agents e.g., bevacizumab	NIH:OVCAR-3 and other cell line xenografts were used for preclinical evaluation of antiangiogenic agents as single agents and in combination with other cytotoxics e.g., paclitaxel	(41, 42, 96)	Clinical evaluation of antiangiogenic agents as single agents and in combination in phase II and phase III trials in ovarian cancer confirmed the preclinical observations (43, 44, 97, 98)
PARP inhibitors (PARPis) e.g., olaparib	Proof of principle in BRCA-deficient cell lines (embryonic stem cells and Chinese hamster cells) and xenografts from these cell lines	(17, 99)	Clinical evaluation of PARP inhibitors in patients with BRCA-associated tumors confirmed the preclinical observations in breast and ovarian cancers (18, 103, 104)
	*In vivo* evaluation in PDX model of BRCA2-associated ovarian cancer and in genetically engineered mouse models of BRCA1 and BRCA2-associated breast cancer	(100–102)	PARPis are currently in phase III clinical trials
Anti-CA125 antibodies e.g., oregovomab, abagovomab	Xenografts with the CA125 positive NIH:OVCAR-3 cell line were used for preclinical evaluation of these agents	(105, 106)	No PFS or OS benefit was detected in large randomized phase III trials for either oregovomab and abagovomab (107, 108)
Anti-HER-2 agents e.g., trastuzumab, pertuzumab	NIH:OVCAR-3, SKOV3, and OVCA433 cell lines and associated xenografts were used for preclinical evaluation of anti-HER-2 drugs as single agents	(109, 110)	Limited single agent activity of trastuzumab and pertuzumab in ovarian cancer (111, 112)
			Improved PFS with pertuzumab and gemcitabine in platinum resistant ovarian cancer (113)

In conclusion, cell lines with confirmed molecular identity using targeted sequencing and copy number profiling may be extremely valuable as *in vitro* models, particularly those with well defined molecular alterations such as BRCA1/2 or PI3K mutations. Xenograft models of established EOC cell lines are still commonly used in preclinical drug development, but are increasingly giving place to PDXs which offer the important advantage of closely resembling original patients’ tumors and adequately capturing the molecular and intratumoral heterogeneity of the original tumors. Finally, genetically engineered mouse models hold promise as they may mimic all major elements of human EOCs including stromal-tumor interactions without the requirement of an immunodeficient background. Clearly, there is no one best preclinical EOC model. Rather, preclinical evaluation of experimental anticancer agents should include multiple model systems in order to increase the possibility of correctly predicting their clinical activity.

## Conflict of Interest Statement

The authors declare that the research was conducted in the absence of any commercial or financial relationships that could be construed as a potential conflict of interest.
